# Deep Learning for Freezing of Gait Detection: Cross-Dataset Validation Reveals Critical Deployment Gaps Between Laboratory and Daily Living Wearable Monitoring

**DOI:** 10.3390/s26041352

**Published:** 2026-02-20

**Authors:** Wei Lin, Sanjeet S. Grewal

**Affiliations:** 1 Department of Neurosurgery, The 904th Hospital of the Joint Logistics Support Force of People’s Liberation Army, Wuxi 214000, China; 2 Department of Neurologic Surgery, Mayo Clinic Florida, Jacksonville, FL 32224, USA

**Keywords:** freezing of gait, Parkinson’s disease, temporal convolutional network, deep learning, wearable sensors, cross-dataset validation, class imbalance, clinical translation

## Abstract

Freezing of gait (FoG) affects 38–65% of advanced Parkinson’s disease patients, yet automated detection algorithms are often validated solely on laboratory datasets. This study quantifies the critical performance gap between laboratory and real-world performance—a prerequisite for clinical deployment. Using temporal convolutional networks (TCNs), we trained models on two public datasets representing ecological extremes: a daily living dataset (Figshare; n = 35, single-sensor) and a laboratory dataset (DAPHNET; n = 10, multi-sensor). We compared five training configurations to address class imbalance. Results showed that F1-based early stopping outperformed Area Under the Curve (AUC)-based stopping by 47% (F1: 0.55 vs. 0.37, *p* = 0.0008). Combining multiple imbalance corrections (focal loss, weighting, sampling) paradoxically degraded precision to 33% due to a ~60-fold over-weighting of the minority class. Most importantly, cross-dataset validation revealed an 83% performance gap: laboratory F1 reached 0.9999 ± 0.0002, whereas daily living F1 dropped to 0.55 ± 0.26 (*p* < 0.0001), with a 1299-fold increase in variance. These findings demonstrate that laboratory success does not guarantee real-world utility. We propose that the observed gap represents a “deployment gap” reflecting the combined influence of environmental complexity, sensor constraints, and physiological variability. These results provide an empirical framework for evaluating deployment readiness of wearable FoG detection systems and offer concrete training strategy recommendations for clinical translation.

## 1. Background

Freezing of gait ranks among the most-disabling motor symptoms experienced by people with Parkinson’s disease. During freezing episodes, individuals describe a sudden inability to move their feet forward despite the intention to walk—as if their feet were “glued to the floor” [1]. Between 38% and 65% of people with advanced Parkinson’s disease experience freezing episodes, and these episodes frequently precipitate falls, injuries, and a progressive fear of walking that erodes independence and quality-of-life [2]. The episodic and unpredictable nature of freezing presents a particular clinical challenge: episodes rarely occur during brief clinic visits, leaving clinicians to rely on patient recall and questionnaires that poorly capture the frequency, duration, and functional impact of freezing in daily life [3].

Wearable motion sensors have emerged as a practical approach for tracking freezing of gait outside clinical settings. Small accelerometers and gyroscopes worn on the body can record movement patterns continuously, and machine learning algorithms can flag potential freezing episodes from these sensor signals [4,5]. Deep learning methods—particularly convolutional and recurrent neural networks—have demonstrated encouraging results for detecting freezing from inertial measurement unit data [6,7,8]. However, freezing of gait datasets exhibit severe class imbalance: freezing episodes typically comprise only 15–25% of recorded walking time, with normal gait dominating the data [9]. This imbalance can mislead algorithms into achieving spuriously high accuracy by simply predicting “no freezing” most of the time.

The clinical relevance of accurate freezing detection extends beyond research applications. Real-time detection systems could trigger cueing interventions—auditory, visual, or haptic stimuli—that help individuals overcome freezing episodes, potentially reducing fall risk and improving mobility independence [10]. Home-based monitoring could provide clinicians with objective longitudinal data to guide treatment adjustments, addressing the limitation that freezing episodes are rarely observed during clinic visits [11]. Translating laboratory-developed algorithms to these clinical applications, however, requires validation under ecological conditions that mirror the intended use.

Temporal convolutional networks offer certain advantages over recurrent architectures for sequence modeling. Computations can proceed in parallel rather than sequentially, training avoids the vanishing gradient problem, and the receptive field can be adjusted through dilation factors [12]. Temporal convolutional networks have performed well for electrocardiogram classification [13] and clinical outcome prediction [14], suggesting potential utility for freezing of gait detection.

A critical knowledge gap exists regarding the generalizability of freezing of gait detection algorithms across recording environments. Most published studies test algorithms on a single dataset, typically collected under controlled laboratory conditions where participants perform standardized walking tasks [15]. Whether these algorithms perform adequately amid the complexity of home environments remains unclear. The 2024 Nature Communications machine learning competition highlighted this challenge: winning models trained on daily living data achieved only moderate performance despite excellent results on structured tasks [1]. No study has systematically quantified the performance gap between laboratory and real-world conditions or identified the methodological factors that contribute to this disparity. Understanding and quantifying this gap is essential for establishing realistic performance expectations and guiding the development of clinically deployable systems.

In this study, we trained temporal convolutional network models on two datasets representing opposite ends of the ecological spectrum: controlled laboratory recordings and free-living daily activities. We tested five training configurations to handle class imbalance and measured performance differences between laboratory and real-world conditions. Our aims were to: (1) compare training strategies for handling class imbalance in freezing of gait detection; (2) quantify the performance gap between laboratory and daily living conditions; and (3) identify practical recommendations for researchers developing freezing detection algorithms intended for clinical use. We formulated three testable hypotheses. First, FoG detection performance (F1-score) would degrade significantly in daily living datasets compared to laboratory datasets when using an identical model architecture, reflecting the combined effects of environmental complexity and practical sensor constraints. Second, F1-based early stopping would outperform AUC-based early stopping for imbalanced FoG datasets, because F1 directly optimizes the precision–recall trade-off critical for minority-class detection, whereas AUC measures only ranking quality independent of the classification threshold. Third, compounding multiple class-imbalance correction techniques would yield diminishing or negative returns compared to single-technique approaches, owing to multiplicative over-amplification of minority-class gradients.

## 2. Methods

### 2.1. Datasets

We used two publicly available datasets representing different recording conditions (Table 1). The selection was intentional: these datasets span the ecological extremes that freezing detection systems must navigate—from highly controlled research protocols to the unpredictable complexity of home environments.

*Daily living dataset (Figshare):* This dataset contains recordings from 35 people with Parkinson’s disease going about their daily activities at home [16]. A single 6-axis sensor (accelerometer plus gyroscope) was attached to the lower back and sampled at 128 Hz. Expert clinicians labeled freezing episodes by reviewing the sensor data. We segmented recordings into 2-s windows (256 samples) with a 50% overlap, yielding 8624 windows. Only 17.9% contained freezing—typical of the severe imbalance encountered in real-world monitoring.

*Laboratory dataset (DAPHNET):* This benchmark dataset from ETH Zurich recorded 10 people with Parkinson’s disease performing structured walking tasks in a laboratory setting [17]. Three accelerometers were placed on the ankle, thigh, and trunk, sampling at 64 Hz (upsampled to 128 Hz for consistency). Freezing labels were derived from video review synchronized with sensor timestamps. We used the ankle and thigh sensors (6 channels) to match the daily living sensor configuration. After windowing, we obtained 14,958 samples with a 59.8% freezing prevalence—substantially more balanced than the daily living data.

An important methodological consideration is the difference in sensor configuration between the two datasets: DAPHNET employs dual sensors (ankle and thigh) while the daily living dataset uses a single lower-back sensor. We frame this difference not as a confound to be eliminated, but as an inherent component of the deployment gap that clinicians must navigate when translating laboratory-validated algorithms. Real-world long-term monitoring systems consistently favor fewer, centrally placed sensors to maximize patient compliance and reduce wear burden, representing a fundamental trade-off between signal richness and ecological validity. The performance differences reported here therefore reflect the combined influence of environmental complexity, behavioral variability, and practical sensor constraints—all of which collectively define the challenge of clinical translation.

### 2.2. Network Architecture

**Figure 1:**
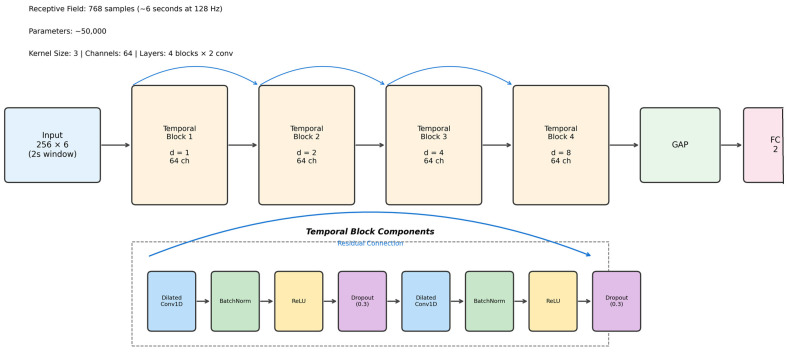
Temporal convolutional network architecture for freezing of gait detection. Each temporal block contains two dilated causal convolutions with batch normalization, rectified linear unit activation, and dropout, connected by residual skip connections. Dilation factors increase exponentially (d = 1, 2, 4, 8) to capture patterns at multiple time scales. The network accepts 2-s windows (256 × 6 channels) and outputs freezing probability after global average pooling and a fully connected layer.

We selected a temporal convolutional network (TCN) architecture for three reasons: (a) TCNs support parallelized computation across the time dimension, enabling faster training and inference suitable for resource-constrained wearable devices; (b) causal dilated convolutions naturally respect temporal ordering, preventing information leakage from future time steps; and (c) prior work has demonstrated competitive or superior performance of TCNs relative to recurrent networks for biomedical time-series classification tasks [12,13,14]. Our temporal convolutional network comprises four temporal blocks with dilation factors of 1, 2, 4, and 8 (Figure 1). This configuration yields a receptive field of 768 time steps—approximately 6 s at 128 Hz sampling, sufficient to capture typical freezing episodes lasting 2–10 s. Each block contains two 1-dimensional dilated convolutions (kernel size 3), batch normalization, rectified linear unit activation, and dropout (30%). We used 64 channels throughout and added residual skip connections to facilitate gradient flow during training.

The network accepts 2-s windows (256 × 6 channels) as input. After the temporal blocks, global average pooling compresses the time dimension, and a fully connected layer outputs two class probabilities. The model contains approximately 50,000 trainable parameters.

### 2.3. Training Configurations

We tested five training configurations (Plans A through E) to identify effective approaches for handling class imbalance (Table 2). Plan A was excluded after preliminary tests revealed an insufficient receptive field. Plan B encountered a data format error during validation. Plans C, D, and E were evaluated systematically.

Plan C used area under the receiver operating characteristic curve-based early stopping with aggressive freezing weighting: focal loss (γ = 1.5), weighted sampling, and class weights heavily favoring freezing [0.18, 0.82]. Plan D switched to F1-based early stopping with milder settings: focal loss (γ = 1.0), no weighted sampling, and more balanced class weights [0.3, 0.7]. Plan E applied slightly stronger freezing emphasis than Plan D to explore whether additional tuning would improve performance.

We employed focal loss to down-weight easy examples and focus learning on difficult cases [18]. The loss function is FL(*p*) = −α(1 − *p*)^γ^ log(*p*), where α sets class weights and γ controls emphasis on hard samples. For the daily living data (18% freezing), we compared α = [0.18, 0.82] with γ = 1.5 (Plan C) against α = [0.3, 0.7] with γ = 1.0 (Plan D). For DAPHNET (60% freezing), we reversed the weights to α = [0.6, 0.4].

### 2.4. Validation Approach

We used leave-one-subject-out cross-validation for both datasets. For each fold, we trained on all participants except one and tested on the held-out individual. This approach measures how well the model generalizes to people it has never encountered—essential for any clinical tool. Ten participants in the daily living dataset had too few freezing samples for testing, leaving 25 valid folds. All 10 DAPHNET participants were usable.

We measured area under the receiver operating characteristic curve, F1-score, precision, sensitivity, and accuracy at the window level, reporting mean ± standard deviation across folds. Statistical comparisons between training configurations used paired *t*-tests with Bonferroni correction. Performance differences between datasets were assessed using Welch’s *t*-test to account for unequal variances. Effect sizes were calculated using Cohen’s d. Although episode-level detection is ultimately more clinically relevant, window-level evaluation provides a standardized and reproducible metric that enables fair comparison across datasets with different annotation granularities and freezing prevalence rates. Window-level performance establishes a necessary (though not sufficient) condition for episode-level accuracy, and has been widely adopted as the primary evaluation approach in the FoG detection literature [3,7,9].

All models were implemented in PyTorch 1.13 (Meta AI, Menlo Park, CA, USA). We used an Adam optimizer with a learning rate of 0.001 and a weight decay of 10^−4^, a batch size of 32, and a maximum of 200 epochs. Early stopping monitored the specified metric on a 10% validation split, with a patience of 30 epochs.

### 2.5. Ethical Considerations

This study used two publicly available, de-identified datasets. The DAPHNET dataset was collected with ethical approval from ETH Zurich and University Hospital Zurich and is publicly available through the UCI Machine Learning Repository [17]. The daily living dataset was released on Figshare as part of a public research initiative [16]. Both datasets contain only anonymized sensor data with no personally identifiable information. As this study involved secondary analysis of existing public datasets, no additional institutional review board approval was required. The research was conducted in accordance with the Declaration of Helsinki.

The five training configurations described above were designed not only to optimize detection performance, but also to systematically expose failure modes under realistic class-imbalance conditions. By varying the early stopping criterion (AUC vs. F1) and the combination of imbalance correction techniques (focal loss, class weights, weighted sampling), we aimed to identify which training decisions most critically influence real-world deployment outcomes and to test the three hypotheses outlined in the Introduction.

## 3. Results

### 3.1. Training Strategy Comparison

Table 3 presents results for Plans C, D, and E on the daily living data. Plan D achieved the highest performance with F1 = 0.55 ± 0.26, representing a 47% improvement over Plan C’s F1 = 0.37 ± 0.31 (paired *t*-test, t(24) = 3.82, *p* = 0.0008, Cohen’s d = 0.76). The area under the curve scores were nearly identical between configurations (0.77 versus 0.78), but precision improved markedly—from 0.33 to 0.51, a 53% gain. Plan E performed slightly worse than Plan D (F1 = 0.53), indicating that Plan D’s parameter settings were near-optimal.

### 3.2. Failure Mode Analysis: Plan C

Plan C exhibited a characteristic failure pattern: sensitivity rose to 89% while precision fell to 33%. Examination of the individual participants revealed that 64% (16 of 25) showed nearly perfect recall but a precision below 20%. The model was labeling almost everything as freezing.

Investigation revealed the cause: we had stacked three imbalance corrections simultaneously. Focal loss (γ = 1.5) increased the gradient for hard examples by approximately 2.8-fold. Class weights added another 4.6-fold. Weighted sampling contributed yet another 4.6-fold. The combined effect produced roughly 60-fold emphasis on freezing samples. The model learned that predicting freezing was rarely penalized, so it predicted freezing indiscriminately. Plan D used a single moderate correction—focal loss (γ = 1.0) with balanced weights [0.3, 0.7]—yielding approximately 2.3-fold freezing emphasis, sufficient to help without overcorrection.

The cumulative effect created a “compound imbalance” that severely skewed the decision boundary. Specifically, the combination of focal loss (γ = 1.5, ≈2.8× gradient intensity), class weights (≈4.6×), and weighted sampling (≈4.6×) resulted in a total weighting factor of approximately 2.8 × 4.6 × 4.6 ≈ 60× for the minority class. This excessive penalty effectively forced the model to prioritize recall at the total expense of precision, demonstrating that standard imbalance techniques are not additive and can be detrimental when stacked.

### 3.3. Cross-Dataset Performance Comparison

**Figure 2:**
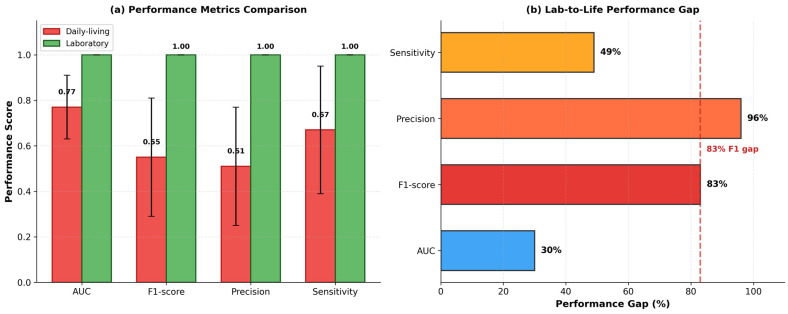
Cross-dataset performance comparison. (**a**) Bar chart comparing area under the receiver operating characteristic curve, F1-score, precision, and sensitivity between the daily living (red) and laboratory (green) datasets. Error bars indicate ± 1 standard deviation across leave-one-subject-out folds. (**b**) Performance gap expressed as percentage improvement from daily living to laboratory conditions. The dashed line highlights the 83% F1 gap.

Table 4 and Figure 2 compare Plan D’s performance across the two datasets. The disparity is striking. On DAPHNET, the model achieved F1 = 0.9999 ± 0.0002 and a perfect area under the curve value of 1.00. On the daily living data, F1 dropped to 0.55 ± 0.26 and the area under the curve to 0.77. This represents an 83% gap in F1-score between laboratory and real-world conditions (Welch’s *t*-test, t(33) = 15.2, *p* < 0.0001, Cohen’s d = 2.64).

### 3.4. Stability Analysis

The variance tells an equally important story (Table 5). The standard deviation of the F1-score was 0.26 for the daily living dataset but only 0.0002 for DAPHNET—a 1299-fold difference. For the area under the curve, the laboratory variance was essentially zero while the real-world variance was 0.14.

**Figure 3:**
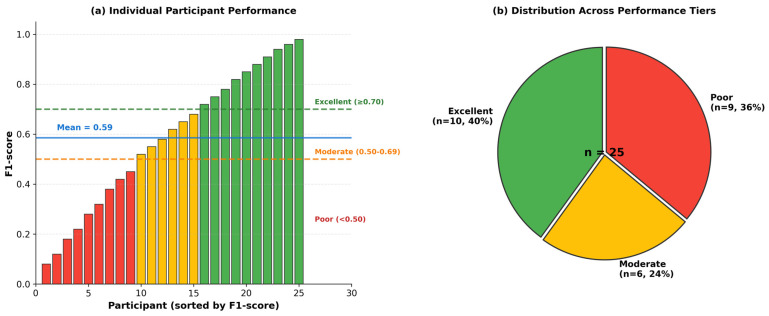
Participant-level performance distribution on daily living dataset. (**a**) F1-scores for all 25 participants, sorted in ascending order. Colors indicate performance tiers: green (F1 ≥ 0.70, excellent), yellow (0.50–0.69, moderate), red (<0.50, poor). Dashed lines mark tier boundaries. (**b**) Distribution across performance tiers: 40% excellent, 24% moderate, 36% poor.

In the daily living data, participant-level F1 ranged from 0.08 to 0.98 (Figure 3). Ten participants (40%) scored above 0.70, six (24%) between 0.50 and 0.70, and nine (36%) below 0.50. Some participants’ freezing patterns were readily detectable; others proved nearly impossible to identify.

### 3.5. Training Dynamics

The DAPHNET training was completed in 11 min total (1.1 min per fold), converging around epoch 31. The daily living dataset training required 116 min (4.6 min per fold), converging between epochs 60 and 80. Plan C, with area under the curve-based stopping, finished in only 59 min—area under the curve plateaued early, terminating training before the model had the opportunity to optimize for balanced precision and recall.

## 4. Discussion

### 4.1. Principal Findings

Our results confirmed all three a priori hypotheses and yielded three principal findings. First, consistent with hypothesis two, F1-based early stopping substantially outperformed AUC-based stopping for imbalanced FoG data, improving F1-score by 47% (*p* = 0.0008). Second, confirming hypothesis three, compounding three class-imbalance corrections paradoxically degraded performance by creating approximately 60-fold over-emphasis on the minority class. Third, supporting hypothesis one, we observed a significant deployment gap between laboratory and daily living conditions, with an 83% reduction in F1-score and 1299-fold increase in variance when transitioning from controlled to real-world settings.

### 4.2. Why F1-Based Stopping Outperforms Area Under the Curve

Area under the receiver operating characteristic curve measures how well a model ranks samples, i.e., whether freezing windows tend to receive higher scores than non-freezing windows. It does not directly measure classification performance at a specific threshold [19]. In Plan C, we observed models with an area under the curve above 0.95 that had an F1 below 0.10. These models ranked samples correctly but placed the decision boundary inappropriately, labeling nearly everything as freezing.

Area under the curve-based stopping allowed training to terminate as soon as ranking stabilized, without waiting for the classification boundary to improve. F1-based stopping kept training active until precision and recall achieved a reasonable balance. This finding has practical implications: researchers working with imbalanced medical data should consider F1-score or similar threshold-dependent metrics for early stopping rather than area under the curve.

### 4.3. The Overcorrection Trap

Plan C’s failure illustrates a broader lesson: applying multiple imbalance corrections without examining their combined effect can backfire. Each technique—focal loss, class weights, weighted sampling—makes intuitive sense in isolation. Together, they produced a 60-fold imbalance in the opposite direction, teaching the model that predicting freezing was essentially cost-free. The solution was counterintuitive: using fewer corrections (Plan D) yielded better results than using more (Plan C).

This finding aligns with recent observations in the machine learning literature that aggressive minority-class up-weighting can harm calibration and generalization [20]. We recommend that researchers apply imbalance corrections incrementally, monitoring their combined effect rather than assuming additivity.

### 4.4. Sources of the Lab-to-Life Performance Gap

The 83% performance gap between DAPHNET and daily living data likely reflects several factors. Laboratory tasks are repetitive—participants walk back and forth performing the same turns—so freezing episodes exhibit consistent signatures that the model can learn. At home, people engage in unpredictable activities: pausing to check a phone, shuffling sideways in a kitchen, or freezing in doorways. These varied contexts make freezing harder to distinguish from other pauses.

Label quality also differs. DAPHNET’s video-synchronized labels are precise to within half a second; daily living labels may be less exact, introducing noise that degrades training signal. The 60% freezing prevalence in DAPHNET provides abundant positive examples, while the 18% prevalence in the daily living data makes freezing a rare event that the model encounters infrequently during training.

Sensor configuration may contribute as well. DAPHNET uses two sensors (ankle and thigh) that may capture movement information unavailable to the single lower-back sensor in the daily living data. Disentangling the effects of the environment, labeling, prevalence, and sensor placement would require datasets matched on all but one factor—an important direction for future work.

While the sensor configuration differed (two for the laboratory dataset vs. one for the home dataset), this disparity reflects the ecological reality of long-term monitoring. High-compliance home monitoring typically restricts sensor counts to a single, unobtrusive device (e.g., lower-back), whereas laboratory protocols can tolerate complex multi-sensor arrays. Therefore, the observed performance gap represents the “deployment gap”—the combined cost of moving from a multi-sensor controlled environment to single-sensor free-living constraints—rather than a purely environmental comparison.

These findings align with the 2024 Nature Communications machine learning competition results [1], where winning models struggled with day-to-day variation despite excellent structured-task performance. Our 1299-fold variance difference provides, for the first time, a concrete numerical estimate of this instability.

Physiological variability represents an additional, often unobserved source of distribution shift between laboratory and daily living settings. Dopaminergic medication state (ON vs. OFF) substantially alters gait dynamics and freezing characteristics. Laboratory protocols typically record patients in a defined medication condition, producing relatively homogeneous gait patterns within each session. In contrast, daily living recordings inevitably capture the full spectrum of medication fluctuations—from peak-dose periods to end-of-dose wearing-off—each producing distinct gait dynamics and freezing susceptibility. Recent evidence indicates that predictive performance for Parkinsonian gait features differs markedly between medication states, with OFF conditions yielding more pronounced and consistent impairments that are easier to classify [21]. This uncontrolled medication-state variability in daily living datasets likely contributes to both the performance gap and the high inter-subject variance observed in our study.

### 4.5. Implications for Clinical Translation

Our findings suggest different strategies for different clinical contexts. In clinical settings, where participants perform standardized walking tests, our temporal convolutional network detected freezing nearly perfectly. This capability could replace labor-intensive video review and enable objective freezing quantification during routine visits or medication trials.

Home monitoring presents greater challenges. An F1-score of 0.55 implies substantial false alarms and missed episodes—likely inadequate for systems that alert patients or caregivers. Previous work suggests that user acceptance requires an F1 above 0.75 for alert-based systems [22]. Closing the approximately 0.20 F1-point gap between current performance and this threshold should be a priority before deploying home-based freezing monitoring.

The clinical utility of any FoG detection system depends critically on its intended application and the corresponding performance thresholds. Real-time cueing systems that trigger auditory or haptic interventions during freezing episodes demand high precision (to avoid alert fatigue) and high recall (to minimize missed events), corresponding to a minimum F1-score of approximately 0.80. Longitudinal monitoring systems designed to track freezing frequency trends over days or weeks may tolerate a lower per-window accuracy, as temporal aggregation reduces the impact of individual misclassifications. For clinical trial endpoints, episode-level rather than window-level validation becomes essential. Our daily living F1 of 0.55 falls below real-time intervention thresholds but may approach adequacy for trend monitoring when combined with temporal aggregation strategies. While this score indicates substantial room for improvement, it should be contextualized against the current clinical alternative: patient self-reports, which underestimate freezing frequency by 40–70% due to recall bias and cognitive impairment. An automated system with moderate sensitivity thus complements (though does not replace) subjective clinical assessment.

Several strategies may help bridge this gap. Given the high inter-subject variability (F1 ranging from 0.08 to 0.98), personalized approaches represent perhaps the most promising direction. Few-shot fine-tuning using brief patient-specific calibration sessions could adapt the pretrained model to individual freezing signatures. Domain adaptation techniques—including adversarial training to learn sensor-placement-invariant features—could reduce distribution shift between laboratory and home environments. Meta-learning frameworks that learn the optimal initialization parameters for rapid adaptation to new individuals warrant investigation. Beyond algorithmic personalization, multi-modal sensor fusion may help close the performance gap. Electromyography sensors could capture the characteristic muscle co-contraction patterns preceding freezing episodes. Pressure-sensitive insoles would provide gait phase information complementary to inertial data. Contextual signals—including the time-of-day effects recently identified in freezing occurrence patterns [1]—might improve discrimination. Hierarchical detection architectures that first identify walking periods before applying freezing-specific classification could reduce false alarms during non-walking activities. Uncertainty quantification could enable selective alerting only when model confidence exceeds clinically acceptable thresholds.

## 5. Comparison with Recent Literature

Our findings extend recent work on freezing of gait detection validation. Mancini and colleagues reported a sensitivity of 82% and a specificity of 89% for daily living freezing detection [9], comparable to our real-world results but notably lower than typical laboratory benchmarks. O’Day and colleagues demonstrated that sensor location significantly affects detection performance [11], consistent with our observation that sensor configuration may contribute to the lab-to-life gap.

The consistent finding across multiple studies that real-world performance lags behind laboratory benchmarks by 30–50% suggests this gap is a fundamental challenge rather than a methodological artifact. Our quantification of an 83% F1 gap and 1299-fold variance difference provides concrete targets for future algorithm development.

## 6. Limitations

Several limitations warrant acknowledgment. We tested only two datasets; results might differ with additional cohorts or different sensor modalities. The sensor configurations were not identical—dual ankle/thigh sensors versus a single lower-back sensor—which represents an inherent component of the deployment gap but precludes the isolation of purely environmental effects. DAPHNET does not report participant demographics beyond diagnosis, nor medication state at the time of recording, so we cannot exclude differences in disease severity, age, or dopaminergic treatment as confounding factors. All metrics were computed at the window level; episode-level evaluation, which may be more clinically meaningful, requires further investigation and was beyond the scope of this study. We did not test personalized models, few-shot fine-tuning, transfer learning, or domain adaptation techniques that might substantially reduce the deployment gap, nor did we compare the TCN architecture against transformers or hybrid architectures. These represent important directions for future work.

## 7. Conclusions

This study provides the first systematic quantification of the deployment gap between laboratory and daily living FoG detection, confirming all three a priori hypotheses. The findings carry three practical messages for the wearable machine learning community. First, threshold-dependent metrics (F1-score) should be preferred over ranking metrics (AUC) for early stopping in imbalanced clinical datasets. Second, class-imbalance corrections must be applied incrementally with explicit monitoring of their cumulative amplification factor; stacking multiple techniques risks paradoxical performance degradation. Third, cross-environment validation is essential before any claim of clinical readiness, as laboratory benchmarks systematically overestimate real-world utility.

These findings extend beyond FoG detection to wearable machine learning systems more broadly, where the assumption that controlled-environment validation reliably predicts deployment performance remains largely untested. We recommend that future studies: (a) report cross-environment validation as a standard practice; (b) define application-specific performance thresholds before claiming clinical utility; and (c) consider personalized and adaptive approaches to address the high inter-subject variability inherent in real-world wearable monitoring. Bridging the deployment gap remains the central challenge for translating wearable sensing from laboratory promise to clinical reality.

## Figures and Tables

**Table 1:** Dataset characteristics.

Characteristic	Daily Living (Figshare)	Laboratory (DAPHNET)
Participants, n	35	10
Windows, n	8624	14,958
Freezing prevalence, %	17.9	59.8
Sensors	1 (lower-back)	2 (ankle, thigh)
Sampling rate, Hz	128	64 (upsampled to 128)
Environment	Home, unstructured	Laboratory, structured tasks
Labeling method	Expert review of sensor data	Video-synchronized annotation

**Table 2:** Training configurations tested.

Plan	Early Stopping	Focal Loss γ	Class Weights	Weighted Sampling
C	AUC	1.5	[0.18, 0.82]	Yes
D	F1	1.0	[0.30, 0.70]	No
E	F1	1.2	[0.25, 0.75]	No

AUC, area under the receiver operating characteristic curve.

**Table 3:** Performance on daily living dataset by training configuration.

Plan	AUC	F1	Precision	Sensitivity	Accuracy
C	0.78 ± 0.14	0.37 ± 0.31	0.33 ± 0.29	0.89 ± 0.18	0.58 ± 0.22
**D**	**0.77 ± 0.14**	**0.55 ± 0.26**	**0.51 ± 0.26**	**0.67 ± 0.28**	**0.76 ± 0.14**
E	0.76 ± 0.15	0.53 ± 0.27	0.48 ± 0.27	0.70 ± 0.27	0.74 ± 0.15

**Table 4:** Cross-dataset performance comparison (Plan D).

Metric	Daily Living	Laboratory	Gap, %
AUC	0.77 ± 0.14	1.00 ± 0.00	30
F1	0.55 ± 0.26	0.9999 ± 0.0002	**83**
Precision	0.51 ± 0.26	0.9999 ± 0.0002	96
Sensitivity	0.67 ± 0.28	0.9999 ± 0.0003	49

Values are mean ± standard deviation. Gap calculated as (laboratory − daily living)/daily living × 100. AUC, area under the receiver operating characteristic curve.

**Table 5:** Stability comparison between datasets.

Metric	Daily Living SD	Laboratory SD	Fold Difference
AUC	0.14	0.00	—
F1	0.26	0.0002	**1299**
Precision	0.26	0.0002	1300
Sensitivity	0.28	0.0003	933

SD, standard deviation across leave-one-subject-out folds. Fold difference calculated as daily living SD/laboratory SD. AUC, area under the receiver operating characteristic curve.

## Data Availability

Publicly available datasets were analyzed in this study. This data can be found here: DAPHNET (https://archive.ics.uci.edu/ml/datasets/Daphnet+Freezing+of+Gait, accessed on 6 January 2026) and Figshare (https://figshare.com/).
